# Supplementary material for: Continuous Growth Monitoring and Prediction with 1D Convolutional Neural Network Using Generated Data with Vision Transformer
Source: Plants (Basel). 2024 Nov 4;13(21):3110. doi: 10.3390/plants13213110 (PMC11548696; doi:10.3390/plants13213110)
Supplement: Supplementary file 1 [file plants-13-03110-s001.zip › plants-3246943-supplementary.pdf]

plants

MDPI

Continuous monitoring and prediction with generated data using vision-transformer and 1D convolutional neural network

Woo-Joo Choi[1], Se-Hun Jang[1], Taewon Moon[2], Kyeong-Su Seo[1], Da-Seul Choi[1] and Myung-Min Oh[1]*

[1]Division of Animal, Horticultural and Food Sciences, Chungbuk National University, Cheongju 28644, Republic of Korea; ujuchoe79@chungbuk.ac.kr; zsh8976@naver.com; nari4491@naver.com; dlfgrhaks@naver.com
[2]Smart Farm Research Center, Korea Institute of Science and Technology (KIST), Gangneung, Gangwon, Republic of Korea
* Correspondence: moh@cbnu.ac.kr.

Supplementary Table S1. The R^2, root mean square error (RMSE), and normalized RMSE (NRMSE) for the trained image converter. The selected models were EfficientNet and ViT-s.

**Output**	**Shoot fresh weight**			**Shoot dry weight**			Leaf area		
**Metrics**	**R^2**	**RMSE**	**NRMSE**	**R^2**	**RMSE**	NRMSE	R^2	RMSE	NRMSE
Convolutional neural network (ConvNets)									
VGG19	−0.25	93.06	0.99	−0.37	4.68	1.05	−0.21	1457.87	0.95
ResNet	0.57	54.43	0.56	0.36	4.68	0.73	−0.21	767.47	0.53
MobileNet	0.31	69.04	0.74	0.11	3.77	0.85	0.46	969.21	0.63
Inception-v3	0.63	50.39	0.47	0.41	3.02	0.6	0.67	747.2	0.44
Xception	0.68	46.99	0.50	0.45	3.01	0.67	0.67	748.72	0.49
EfficientNet	0.83	33.94	0.36	0.51	2.77	0.62	0.85	505.65	0.33
Vision Transformer (ViT)									
ViT	0.83	34.53	0.36	0.55	2.67	0.57	0.83	546.41	0.39
ViT-s	0.88	29.61	0.32	0.64	2.41	0.54	0.90	414.19	0.27
Swin	0.74	42.37	0.45	0.78	1.85	0.42	0.91	402.82	0.26
Conv-mixer	0.82	35.34	0.38	0.7	2.17	0.49	0.89	441.27	0.29

Supplementary Table S2. The R^2, root mean square error (RMSE), and normalized RMSE (NRMSE) for the trained growth predictor. The selected models were 1D ConvNet with U-Net feature extraction layer.

**Structures**	**SFW**			**LA**			**SDW**		
	**R^2**	**RMSE**	**NRMSE**	**R^2**	**RMSE**	**NRMSE**	**R^2**	**RMSE**	**NRMSE**
LSTM	0.55	42.63	0.61	0.56	682.01	0.58	0.0.50	1.6	0.24
Bi-LSTM	0.56	42.17	0.60	0.52	707.61	0.6	0.47	1.64	0.25
1D ConvNet	0.86	23.64	0.34	0.87	366.36	0.31	0.85	0.86	0.13
1D ConvNet with U-Net	0.93	17.28	0.25	0.92	285.93	0.24	0.92	0.64	0.09
